# Has avian influenza virus H9 originated from a bat source?

**DOI:** 10.3389/fvets.2023.1332886

**Published:** 2024-01-08

**Authors:** Kobey Karamendin, Aidyn Kydyrmanov, Sasan Fereidouni

**Affiliations:** ^1^Laboratory of Viral Ecology, Scientific and Production Center of Microbiology and Virology, Department of Virology, Almaty, Kazakhstan; ^2^Research Institute of Wildlife Ecology, University of Veterinary Medicine Vienna, Vienna, Austria

**Keywords:** influenza A, subtype, bat, bird, Kazakhstan, Caspian

## Abstract

Influenza A viruses are important pathogens that can cause diseases with high mortality in humans, animals, and birds; and wild birds are considered the primary reservoir of all subtypes in nature. After discovering the H9 influenza A viruses in bats, questions arose about their potential to serve as an additional natural reservoir and about the priority of the viral origin: Did the virus initially circulate in bats and then transmit to birds or vice versa? Influenza A viruses of the H9 subtype are of particular interest because fatal infections of humans caused by H5, H7, and H10 influenza viruses contained RNA segments from H9 viruses. Recently, a novel subtype of influenza A virus (H19) was reported and it was closely related to the H9 bat influenza A virus by its hemagglutinin structure. The genome of novel H19 has revealed a mixed characteristic genomic signature of both avian and bat influenza viruses. The time to most recent common ancestor (TMRCA) estimates have shown that the divergence time between the bat and avian H9-similar influenza virus occurred approximately at the end of the XVIII century. This article discusses the evolution and possible origin of influenza viruses of the H9 subtype isolated from bats and birds. The obtained data, along with the known data, suggest that the primary reservoir of the H9 influenza virus is wild birds, from which the virus was transmitted to bats. We hypothesize that the novel H19 could be a descendant of an intermediate influenza virus that was in the transition stage of spillover from avian to bat hosts.

## Introduction

Influenza A, as one of the most essential zoonotic infections, continues to pose a significant threat to human health and poultry production worldwide. Historically, wild birds were considered the natural reservoir for avian influenza viruses of all the known subtypes, serving as a central component for viral evolution and transmission to domestic birds and mammals (1). However, influenza A viruses (IAV) phylogenetically distant from avian isolates have been discovered in bats in South America (2, 3), raising questions about the existence of another reservoir in nature and the origin of influenza viruses from either bats or birds (4). In 2016 and 2017, influenza viruses of the H9 subtype were isolated from African bats, phylogenetically very close to the avian cluster (5, 6). It has been shown that despite the high degree of separation of bat populations, some genetic interaction between influenza viruses of the H9 subtype from birds and bats is possible.

Recently, a putatively novel subtype of influenza A virus (H19), closely related to the bat H9 influenza A virus, was reported from a wild duck in the Caspian seashore in Kazakhstan (7). Sequencing of its hemagglutinin (HA) gene (7) has shown the dual nature of its genome, characteristic of both avian and bat influenza viruses. The discovery of this virus has helped contribute to investigations into the genetic interaction between avian and bat influenza viruses and the origin of the H9 subtype from birds or bats.

## Materials and methods

**Figure 1:**
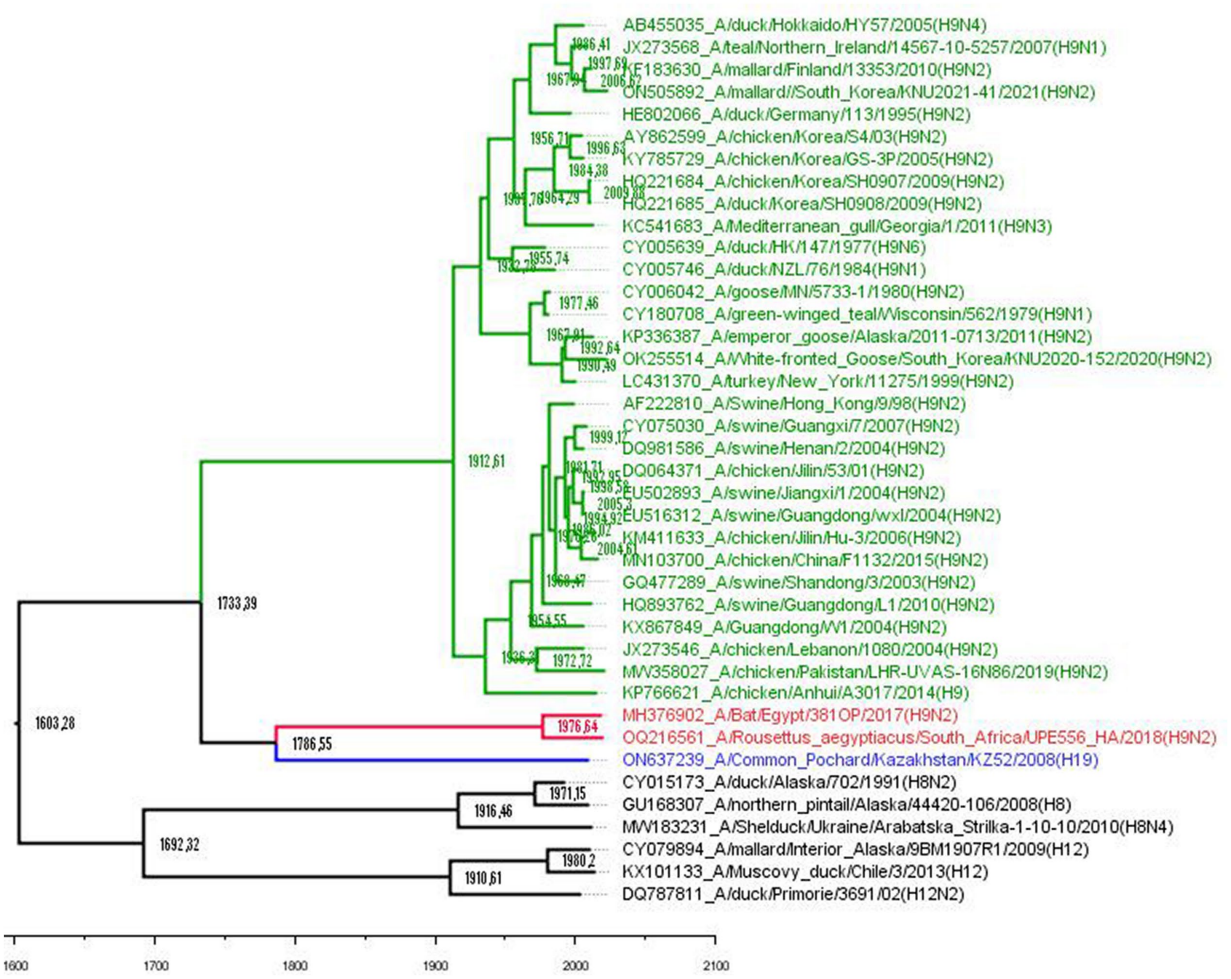
Phylogenetic tree based on the aligned H8, H9, H12, and H19 HA gene nucleotide sequences. Avian H9 subtype viruses are coded with green, bat H9 with red, and H19 with blue. Outgroup is black. A time-measured phylogeny using the BEAST v. 2.7.5 package based on the tip dates of each strain. The substitution model GTR with estimated base frequencies, four gamma categories, and invariant sites (GTR + F + I + G4) was applied. A fast relaxed clock with lognormal distribution was chosen, and the tree prior was set to Coalescent Skyline. The scale bar indicates the years before the most recent sampling time (2021).

A total of 34 HA gene sequences of H9 IAVs representing various hosts (avian, bat, swine, and humans) from Europe, Asia, New Zealand, and North America, along with the novel H19 subtype, were compiled from GenBank. The sequences of influenza viruses of H8 and H12 subtypes were used as outgroups (Figure 1). Nucleotide sequence alignments were created using the Clustal Omega incorporated in the Geneious software package (8). The most appropriate substitution estimation model for use in phylogenetic and molecular clock analyses was recommended by ModelTest, implemented in MEGA 11.0 (9), based on Akaike Information Criterion and Bayesian Information Criterion. Potential N-glycosylation sites (PGS) of the HA were predicted using the NetNGlyc 1.0 server.^1^

To be consistent with previous comparative molecular clock analyses of avian and bat H9 influenza viruses (10), the same models were used: GTR with four gamma categories and invariant sites along with the fast relaxed lognormal molecular clock. A Coalescent Skyline was set as a tree prior. Bayesian time-scaled analyses were conducted by the Bayesian Markov Chain Monte Carlo (BMCMC) method implemented in the BEAST v2.7.5 program (11) utilizing an HA gene sequence dataset. The tests were run for 10 × 10^6^ generations, sampling every 1,000 steps. The convergence of output files was estimated in TRACER v1.7.2, where statistical uncertainty was provided by the 95% HPD values (Supplementary Table S1). The maximum clade credibility tree was generated using Tree Annotator v 2.7.5 and visualized in FigTree v1.4.4.

## Results

The tMRCA estimates show that the divergence between avian and bat H9-like influenza virus subtypes occurred around 1733 CE (Figure 1), in agreement with a previous report that established the time of the MRCA for them as approximately 1700 CE (10). Then, the mixed bat H9-like precursor diverged to H9 bat and H9-like H19 avian influenza viruses around 1786 CE, which still circulate in their respective hosts. Maximum clade credibility trees revealed that H9, H8, and H12 have evolved from ancestors that existed approximately around the 1600s and these data are close to previously estimated data using a host-specific molecular clock (12).

To follow this evolutionary process, it was important to understand the differences in the amino acid structure of HA, related to the bird or bat host specificity. It was established that amino acid substitutions at positions E190D and G225D for H1N1 and Q226L with G228S for H2N2 and H3N2 (H3-numbering) play a key role in adaptation from avian to human host (13, 14). Both bat H9 and avian H19 have avian residues E190, G225, and G228, but avian H19 has a substitution Q226I, while Bat H9 has avian Q226. Analysis of the amino acid consensus sequences of HA from bat H9, avian H19, avian H9, swine, and human viruses revealed the presence of presumably bat-specific sites (highlighted in gray in Table 1).

**Table 1:** Comparison of consensus amino acid sequences of H19 and avian, bat, and mammalian H9 HAs (H9 numbering).

Host	Receptor-binding sites										Bat influenza H9-specific sites							
	166	191	197	198	232	234	235	236	399		5	14	15	17	18	28	32	35
Bat	N	H	E	Q	G	Q/R	G	R	K		K	Q	I	K/R	G	N	D	N
Avian H19	N	N	E	Q	G	Q	G	R	K		A	S	P	K	A	T	D	N
Avian H9	N/G	H	E/V/A	Q	G	Q/I	G	R	K/R		S/A/T	T/G	V/A/T	N	A	T	E	D
Swine	N	H	V	Q	G	Q	G	R	K		S	T	V	N	A	T	E	D
Human	N	H	V	Q	G	Q	G	R	K		P	T	A	N	A	T	E	D
Host	Bat influenza H9-specific sites																	
	38	40	46	47	52	53	54	58	61	62	63	64	66	69	71	72	74	75
Bat	I	S	S	S	E	S	S	L	S	N	E	K	S	V	T	S/N	L	V
Avian H19	I	N	Q	S	H	Q	E	L	S	T	S	K	S	E	D	K	K	I
Avian H9	T/V/I	N/T	H	A/T	H	E	E	M	A	T	N/D	L/Q	R/H	I	D	T	T	I/V
Swine	T	N	H/Q	A	H	E	E	M	A	T	N	L	H	I	D	T	T	I
Human	T	N	Q	A	H	E	E	M	A	T	N	L	H	I	D	T	T	I
Host	Bat influenza H9-specific sites																	
	84	87	90	102	104	107	112	113	126	127	133	137	139	166	173	177	181	182
Bat	Q	S	D/N	N	T	V	K	I	I	E	V	Q	V	T	E	R	G/K	H
Avian H19	E	L	G	T	Q	I	N	I	S	S	V	P	V	S	E	K	S	D
Avian H9	S	L	G	S	V/I	M/L	N/R	V	A/S	S/R	Q/L	P	I/T	N/S	D	T	G	K
Swine	S	L	G	S	V	M/L	N	V	A	S	Q	P	I	N	D	T	G	K
Human	S	L	G	S	V	M	N	V	A	S	Q	P	I	N	D	T	G	K
Host	Bat influenza H9-specific sites																	
	197	200	208	220	224	243	244	252	255	269	273	280	281	282	283	289	294	300
Bat	S	N/S	S	S	A	G	I	K	T	K	Y	E	T	P	E	E	F	S
Avian H19	S	E	T	S	T	G	I	K	T	R	H	A	AQ	P	L	E	K	S
Avian H9	T	T/M	T	T/V	V	S	V	R	S	S	H	D	L	K/N/S	S	Q	K/R	T
Swine	T	T	T	T	L/V	S	V	R	S	S	H	D	L	N	S	Q	R	T
Human	T	T	T	T	V	S	V	R	S	S	H	D	L	N	S	Q	R	T
Host	Bat influenza H9-specific sites																	
	313	319	320	324	327	335	336	337	367	368	376	383	395	401	405	413	415	418
Bat	Y	K	T	R	T	I	Q	T	A	E	Q	L	N	F	G	Q	I	S/L
Avian H19	N	R	T	K	L	K	E	T	S	E	L	I	D	Y	G	T	I	M
Avian H9	N/N	R/G	V	K	V	K/T/R/A	S	D/S/G	D	Q	R/K	V/I	D	Y	D	A/T	L	M
Swine	N	G	V	K	V	R	S/L	S	D	Q	R	I	D	Y	D	T	L	M
Human	N	G	V	K	V	R	S	S	D	Q	R	I	D	Y	D	T	L	M
Host	Bat influenza H9-specific sites																	
	426	429	438	454	458	463	466	469	472	477	479	483	485	496	498	501	518	519
Bat	V	L	I	R/K	D	S	E	K	D	Q	L	N	E	I/M	S	E	E	N
Avian H19	I	I	V	R	E	S	E	I	G	E	L	N	S	S	Y	S	S	N
Avian H9	I	I	V	N	N	A	S	I/M/R/V	G	E	Y	D	Q	D/N	Q/R	Q/K	S	E
Swine	I	I	V	N	N	A	S		G	E	Y	D	Q	N	R	K	S	E
Human	I	I	V	N	N	A	S		G	E	Y	D	R	N	G	K	S	E
Host	Bat influenza H9-specific sites						NGlyc sites										Cleavage site	
	525	538	545	546	557		29	105	141	192	218	248	298	305	492		333	
Bat	M	T	F	I	T		NSTD	–	NVTY	–	–	–	NTSL	NISK	NGTY		PAIQTR	
Avian H19	L	L	M	V	T		NSTD	–	NVTY	NPSS	–	NQTL	NASL	NISK	NGTY		PIKETR	
Avian H9	L	A	M/L	F	I/N		NSTE	NG(T/M/L)C	NV(T/S)Y	–	NRTF	–	NTTL	N(V/I)SK	NGTY		PA(R/A)SDR	
Swine	L	A	L	F	N		NSTE	–	NVSY	–	NRTF	–	NTTL	NVSK	NGTY		PAR(S/L)SR	
Human	L	A	L	F	N		NSTE	–	NVTY	–	NR(A/T)F	–	NTTL	NVSK	NGTY		PARSSR	

A total of 103 putative bat-specific sites that distinguish bat and avian HAs were identified. Interestingly, avian H19 shared 34 bat-specific positions with bat H9, which confirms the mixed avian and bat nature of the H19 HA genome. The results of the analysis showed that the H19 subtype could have served as an intermediate link between avian and bat influenza virus hosts in the past.

Analysis of the receptor binding site (RBS) of H19, bat and other mammalian H9 HAs has shown their potential avian origin. Six potential PGSs at positions 29, 105, 141, 218, 298, and 305 in HA1 and one 492 in HA2 (H9-numbering) were identified in avian, swine, and human H9 viruses. Two PGSs at positions 192 and 248 were unique for the H19 virus. Some avian isolates had one more PGS at position 105 that was absent in bat and mammalian sequences as well as in the H19 subtype. The HA cleavage site of all avian and mammalian influenza viruses contained one or two basic amino acids that are typical mostly of low pathogenic strains. Interestingly, the cleavage site pattern of novel H19 was defined as PIKETR↓GLF and was identical to that of the bat influenza strain A (H18N11) isolated in South America (2).

## Discussion

Historically, wild birds have been considered the main and the only reservoir of IAV in nature (1). The bat H17 and H18 influenza A subtypes are thought to have diverged from avian lineages in the 14th century (10), and because of their huge difference from conventional IAVs, it is difficult to hypothesize their relationship with avian genotypes. On the contrary, the H9 bat IAVs turned out to be phylogenetically close to avian viruses (5, 6). Mortality cases in humans caused by H5, H7, and H10 IAV subtypes containing H9N2 segments in their genome were registered in Asia (15). The H9 subtype is known for its broad host range, including birds, mammals, and humans, and it is well-established that reassortment between isolates from different host species can generate viruses with pandemic potential (16). Due to the high host adaptability of this subtype, it possessed substantial potential to be transmitted to a new bat host. Obviously, a separate mammalian lineage of the H9 influenza A virus has emerged in bat populations. Interestingly, this H9 subtype lineage emerged in bats, and there is no evidence of similar events in human or pig populations, although it may potentially happen in the future. Extensive circulation of the bat lineage was serologically confirmed by the detection of neutralizing antibodies to H9 in several bat populations in Africa (5). The adaptation process in bats did not affect RBS as nearly all key genetic signature sequences retained avian features. It has been shown that although bats are resistant to avian H9 viruses, they are susceptible to specific bat H9 isolates (17).

The Caspian region is an important place where many wild bird migration routes intersect and different viral lineages and genotypes may potentially mix. In this region, subtypes of influenza A virus H14 (18) and H16 (19) were first discovered and reassortant American lineage H11 (20) and a novel paramyxovirus APMV-20 (21) were first identified. It is possible that mixed avian-bat viruses, such as H19, circulate in Africa, where H9 has been isolated from bats (5, 6), and spread to other places by migrating birds. A case of transmission of the H5 virus from Africa across the Caspian Sea by flamingo was previously described (22).

We assume that bat H9 viruses originated from avian H9 and the new avian H19 subtype could potentially be a descendant of an ancestor virus that further diverged into two lineages—bat and avian.

If we try to answer the question from the title of the article “Has avian influenza virus H9 originated from a bat source?,” based on the findings obtained in this study and available data (5, 6), we are inclined to hypothesize the opposite, that the H9 bat influenza virus originated from an avian reservoir.

With the increasing infection of humans with H9N2 in Asia, a major concern arises that this subtype may be the cause of the next worldwide pandemic, although no human-to-human transmission has yet been reported (4, 23). Therefore, regular monitoring of circulation and evolution of the H9 subtype viruses in animal and human populations have great importance in ensuring preparedness for adverse pandemic scenarios.

## Data availability statement

The datasets presented in this study can be found in online repositories. The names of the repository/repositories and accession number(s) can be found in the article/Supplementary material.

## Ethics statement

The animal study was approved by the Institutional Review Board: The SPC of Microbiology and Virology Local Ethics Committee (Approval number: 02–09-106). The study was conducted in accordance with the local legislation and institutional requirements.

## Author contributions

KK: Formal analysis, Funding acquisition, Investigation, Methodology, Project administration, Writing – original draft, Writing – review & editing. AK: Resources, Supervision, Writing – review & editing. SF: Validation, Writing – review & editing.

## Footnotes

^1^ https://services.healthtech.dtu.dk/services/NetNGlyc-1.0
