# Supplementary material for: Has avian influenza virus H9 originated from a bat source?
Source: Front Vet Sci. 2024 Jan 8;10:1332886. doi: 10.3389/fvets.2023.1332886 (PMC10801046; doi:10.3389/fvets.2023.1332886)
Supplement: Supplementary file 1 [file Table_1.docx]

Supplementary Table S1. The trace data generated by Bayesian MCMC run.

| Summary Statistic | posterior |
| --- | --- |
| mean | -21111.9152 |
| stderr of mean | 1.1193 |
| stdev | 15.6083 |
| variance | 243.6179 |
| median | -21110.9696 |
| value range | [-21180.8214, -21062.4135] |
| geometric mean | n/a |
| 95% HPD interval | [-21143.2434, -21083.0223] |
| auto-correlation time (ACT) | 1.1572E5 |
| effective sample size (ESS) | 194.4 |
| number of samples | 22501 |
