# Supplementary material for: Has avian influenza virus H9 originated from a bat source?
Source: Front Vet Sci. 2024 Jan 8;10:1332886. doi: 10.3389/fvets.2023.1332886 (PMC10801046; doi:10.3389/fvets.2023.1332886)
Supplement: Supplementary file 2 [file Table_2.docx]

Genbank Accession numbers used in the article

| **Accession #** | **Virus Strain** | **Link to Genbank** |
| --- | --- | --- |
| ON637239 | Influenza A virus A/Common Pochard/Kazakhstan/KZ52/2008(H19) | <https://www.ncbi.nlm.nih.gov/nucleotide/ON637239> |
| MH376902 | Influenza A virus A/Bat/Egypt/381OP/2017(H9N2) | <https://www.ncbi.nlm.nih.gov/nucleotide/MH376902> |
| OQ216561 | Influenza A virus A/Rousettus aegyptiacus/South Africa/UPE556_HA/2018(H9N2) | <https://www.ncbi.nlm.nih.gov/nucleotide/OQ216561> |
| AB455035 | Influenza A virus A/duck/Hokkaido/HY57/2005(H9N4) | <https://www.ncbi.nlm.nih.gov/nucleotide/AB455035> |
| JX273568 | Influenza A virus A/teal/Northern Ireland/14567-10-5257/2007(H9N1) | <https://www.ncbi.nlm.nih.gov/nucleotide/JX273568> |
| KF183630 | Influenza A virus A/mallard/Finland/13353/2010(H9N2) | <https://www.ncbi.nlm.nih.gov/nucleotide/KF183630> |
| ON505892 | Influenza A virus A/Mallard(Anas platyrhynchos)/South Korea/KNU2021-41/2021(H9N2) | <https://www.ncbi.nlm.nih.gov/nucleotide/ON505892> |
| HE802066 | Influenza A virus A/duck/Germany/113/1995(H9N2) | <https://www.ncbi.nlm.nih.gov/nucleotide/HE802066> |
| AY862599 | Influenza A virus A/chicken/Korea/S4/03(H9N2) | <https://www.ncbi.nlm.nih.gov/nucleotide/AY862599> |
| KY785729 | Influenza A virus A/chicken/Korea/GS-3P/2005(H9N2) | <https://www.ncbi.nlm.nih.gov/nucleotide/KY785729> |
| HQ221684 | Influenza A virus A/chicken/Korea/SH0907/2009(H9N2) | <https://www.ncbi.nlm.nih.gov/nucleotide/HQ221684> |
| HQ221685 | Influenza A virus A/duck/Korea/SH0908/2009(H9N2)) | <https://www.ncbi.nlm.nih.gov/nucleotide/HQ221685> |
| KC541683 | Influenza A virus A/Mediterranean gull/Georgia/1/2011(H9N3) | <https://www.ncbi.nlm.nih.gov/nucleotide/KC541683> |
| CY005639 | Influenza A virus A/duck/HK/147/1977(H9N6) | <https://www.ncbi.nlm.nih.gov/nucleotide/CY005639> |
| CY005746 | Influenza A virus A/duck/NZL/76/1984(H9N1) | <https://www.ncbi.nlm.nih.gov/nucleotide/CY005746> |
| CY006042 | Influenza A virus A/goose/MN/5733-1/1980(H9N2) | <https://www.ncbi.nlm.nih.gov/nucleotide/CY006042> |
| CY180708 | Influenza A virus (A/green-winged teal/Wisconsin/562/1979(H9N1) | <https://www.ncbi.nlm.nih.gov/nucleotide/CY180708> |
| KP336387 | Influenza A virus (A/emperor goose/Alaska/2011-0713/2011(H9N2) | <https://www.ncbi.nlm.nih.gov/nucleotide/KP336387> |
| OK255514 | Influenza A virus (A/White-fronted Goose/South Korea/KNU2020-152/2020(H9N2) | <https://www.ncbi.nlm.nih.gov/nucleotide/OK255514> |
| LC431370 | Influenza A virus (A/turkey/New York/11275/1999(H9N2) | <https://www.ncbi.nlm.nih.gov/nucleotide/LC431370> |
| AF222810 | Influenza A virus (A/swine/Hong Kong/9/98(H9N2) | <https://www.ncbi.nlm.nih.gov/nucleotide/AF222810> |
| CY075030 | Influenza A virus (A/swine/Guangxi/7/2007(H9N2) | <https://www.ncbi.nlm.nih.gov/nucleotide/CY075030> |
| DQ981586 | Influenza A virus (A/swine/Henan/2/2004(H9N2) | <https://www.ncbi.nlm.nih.gov/nucleotide/DQ981586> |
| DQ064371 | Influenza A virus (A/chicken/Jilin/53/2001(H9N2) | <https://www.ncbi.nlm.nih.gov/nucleotide/DQ064371> |
| EU502893 | Influenza A virus (A/swine/Jiangxi/1/2004(H9N2) | <https://www.ncbi.nlm.nih.gov/nucleotide/EU502893> |
| EU516312 | Influenza A virus (A/swine/Guangdong/wxl/2004(H9N2) | <https://www.ncbi.nlm.nih.gov/nucleotide/EU516312> |
| KM411633 | Influenza A virus (A/chicken/Jilin/Hu-3/2006(H9N2) | <https://www.ncbi.nlm.nih.gov/nucleotide/KM411633> |
| MN103700 | Influenza A virus (A/chicken/China/F1132/2015(H9N2) | <https://www.ncbi.nlm.nih.gov/nucleotide/MN103700> |
| GQ477289 | Influenza A virus (A/swine/Shandong/3/2003(H9N2) | <https://www.ncbi.nlm.nih.gov/nucleotide/GQ477289> |
| HQ893762 | Influenza A virus (A/swine/Guangdong/L1/2010(H9N2) | <https://www.ncbi.nlm.nih.gov/nucleotide/HQ893762> |
| KX867849 | Influenza A virus (A/Guangdong/W1/2004(H9N2) | <https://www.ncbi.nlm.nih.gov/nucleotide/KX867849> |
| JX273546 | Influenza A virus (A/chicken/Lebanon/1080/2004(H9N2) | <https://www.ncbi.nlm.nih.gov/nucleotide/JX273546> |
| MW358027 | Influenza A virus (A/chicken/Pakistan/LHR-UVAS-16N86/2019 (H9N2) | <https://www.ncbi.nlm.nih.gov/nucleotide/MW358027> |
| KP766621 | Influenza A virus (A/chicken/Anhui/A3017/2014 (H9) | <https://www.ncbi.nlm.nih.gov/nucleotide/KP766621> |
| CY015173 | Influenza A virus A/duck/Alaska/702/1931(H8N2) | <https://www.ncbi.nlm.nih.gov/nucleotide/CY015173> |
| GU168307 | Influenza A virus A/northern pintail/Alaska/44420-106/2008(H8) | <https://www.ncbi.nlm.nih.gov/nucleotide/GU168307> |
| MW183231 | Influenza A virus A/shelduck/Ukraine/Arabatska_Strilka-1-10-10/2006(H8N4) | <https://www.ncbi.nlm.nih.gov/nucleotide/MW183231> |
| CY079894 | Influenza A virus (A/mallard/Interior Alaska/9BM1907R1/2009(H12) | <https://www.ncbi.nlm.nih.gov/nucleotide/CY079894> |
| KX101133 | Influenza A virus (A/Muscovy duck/Chile/3/2013(H12) | <https://www.ncbi.nlm.nih.gov/nucleotide/KX101133> |
| DQ787811 | Influenza A virus (A/duck/Primorie/3691/02(H12N2) | <https://www.ncbi.nlm.nih.gov/nucleotide/DQ787811> |
